# Multiscale techniques for parabolic equations

**DOI:** 10.1007/s00211-017-0905-7

**Published:** 2017-07-20

**Authors:** Axel Målqvist, Anna Persson

**Affiliations:** Department of Mathematical Sciences, Chalmers University of Technology and University of Gothenburg, Göteborg, Sweden

**Keywords:** 35K05, 35K58, 65M60

## Abstract

We use the local orthogonal decomposition technique introduced in Målqvist and Peterseim (Math Comput 83(290):2583–2603, 2014) to derive a generalized finite element method for linear and semilinear parabolic equations with spatial multiscale coefficients. We consider nonsmooth initial data and a backward Euler scheme for the temporal discretization. Optimal order convergence rate, depending only on the contrast, but not on the variations of the coefficients, is proven in the $L_\infty (L_2)$-norm. We present numerical examples, which confirm our theoretical findings.

## Introduction

In this paper we study numerical solutions to parabolic equations with highly varying coefficients. These equations appear, for instance, when modeling physical behavior in a composite material or a porous medium. Such problems are often referred to as *multiscale problems*.

Convergence of optimal order of classical finite element methods (FEMs) based on continuous piecewise polynomials relies on at least spatial $H^2$-regularity. More precisely, for piecewise linear polynomials, the error bound depends on $\Vert u\Vert _{H^2}$, which may be proportional to $\epsilon ^{-1}$ if the diffusion coefficient varies on a scale of $\epsilon$. Thus, the mesh width *h* typically must fulfill $h<\epsilon$ to achieve convergence. However, this is not computationally feasible in many applications. To overcome this issue, several numerical methods have been proposed, see, for example, [2, 8, 13, 15, 16, 19], and references therein. In particular, [15, 16] consider linear parabolic equations.

In [13] a generalized finite element method (GFEM) was introduced and convergence of optimal order was proven for elliptic multiscale equations. The method builds on ideas from the variational multiscale method [8, 10], which is based on a decomposition of the solution space into a (coarse) finite dimensional space and a residual space for the fine scales. The method in [13], often referred to as local orthogonal decomposition, constructs a generalized finite element space where the basis functions contain information from the diffusion coefficient and have support on small vertex patches. With this approach, convergence of optimal order can be proved for an arbitrary positive and bounded diffusion coefficient. Restrictive assumptions such as periodicity of the coefficients or scale separation are not needed. Some recent works [1, 6, 7, 14] show how this method can be applied to boundary value problems, eigenvalue problems, semilinear elliptic equations, and linear wave equations.

In this paper we apply the technique introduced in [13] to parabolic equations with multiscale coefficients. We use the diffusion coefficient to construct a generalized finite element space and for the discretization of the temporal domain we use the backward Euler scheme. Using tools from classical finite element theory for parabolic equations, see, e.g, [11, 12, 18], and references therein, we prove convergence of optimal order in the $L_\infty (L_2)$-norm for linear and semilinear equations under minimal regularity assumptions and nonsmooth initial data. The analysis is completed with numerical examples that support our theoretical findings.

In Sect. 2 we describe the problem formulation and the assumptions needed to achieve sufficient regularity of the solution. Section 3 describes the numerical approximation and presents the resulting GFEM. In Sect. 4 we prove error bounds and in Sect. 5 we extend the results to semilinear parabolic equations. Finally, in Sect. 6 we present some numerical examples.

## Problem formulation

We consider the parabolic problem

2.1 $$\begin{aligned} \begin{array}{lll} c\dot{u} - \nabla \cdot (A\nabla u) = f, &{}\quad \, \text {in } \Omega \times (0,T], \\ u = 0, &{}\quad \,\text {on } \partial \Omega \times (0,T], \\ u(\cdot ,0) = u_0, &{}\quad \, \text {in } \Omega , \end{array} \end{aligned}$$

where $T>0$ and $\Omega$ is a bounded polygonal/polyhedral domain in $\mathbb {R}^d$, $d\le 3$. We assume $c=c(x)$, $A = A(x)$, and $f=f(x,t)$. Here we allow both $c$ and *A* to be multiscale (in space), but independent of the time variable.

We let $H^1(\Omega )$ denote the classical Sobolev space with norm

$$\begin{aligned} \Vert v\Vert ^2_{H^1(\Omega )} = \Vert v\Vert ^2_{L_2(\Omega )} + \Vert \nabla v\Vert ^2_{L_2(\Omega )} \end{aligned}$$

and $V=H^1_0(\Omega )$ the space of functions in $H^1(\Omega )$ that vanishes on $\partial \Omega$. We use $H^{-1}(\Omega )$ to denote the dual space to *V*. Furthermore, we use the notation $L_p(0,T;X)$ for the Bochner space with finite normwhere *X* is a Banach space equipped with norm $\Vert \cdot \Vert _X$. Here $v\in H^1(0,T;X)$ means $v,\dot{v}\in L_2(0,T;X)$. The dependence on the interval [0, *T*] and the domain $\Omega$ is frequently suppressed and we write, for instance, $L_2(L_2)$ for $L_2(0,T;L_2(\Omega ))$. Finally, we abbreviate the $L_2$-norm $\Vert \cdot \Vert := \Vert \cdot \Vert _{L_2(\Omega )}$ and define $\mathopen {|||} \cdot \mathclose {|||} :=\Vert A^{1/2}\nabla \cdot \Vert$.

To ensure existence, uniqueness, and sufficient regularity, we make the following assumptions on the data.

We assume

1. $$A\in L_\infty (\Omega ,{\mathbb {R}}^{d \times d})$$, symmetric, and
  $$\begin{aligned} 0<\alpha _1&:={{\mathrm{ess\,inf}}}_{x \in \Omega } \inf _{v \in {\mathbb {R}}^d\setminus \{0\}} \frac{A(x) v \cdot v}{v \cdot v},\\ \infty > \alpha _2&:={{\mathrm{ess\,sup}}}_{x \in \Omega } \sup _{v \in {\mathbb {R}}^d\setminus \{0\}} \frac{A(x) v \cdot v}{v \cdot v}, \end{aligned}$$
2. $$c\in L_\infty (\Omega ,{\mathbb {R}})$$ and
  $$\begin{aligned} 0< \gamma _1 := {{\mathrm{ess\,inf}}}_{x \in \Omega } c(x) \le {{\mathrm{ess\,sup}}}_{x \in \Omega } c(x) =: \gamma _2 < \infty , \end{aligned}$$
3. $$u_0 \in L_2$$,
4. $$f,\dot{f} \in L_\infty (L_2)$$.

We let $(u,v)=\int _{\Omega }uv$ denote the classical $L_2$ inner product and define

$$\begin{aligned} (\cdot , \cdot )_c:= (c\, \cdot , \cdot ). \end{aligned}$$

Due to (A2) this is an inner product and the induced norm $\Vert c^{1/2} \cdot \Vert$ is equivalent to the classical $L_2$-norm.

We emphasize that throughout this work *C* denotes a constant that may depend on the bounds $\alpha _1$ and $\alpha _2$ (often through the contrast $\alpha _2/\alpha _1$), the bounds $\gamma _1$ and $\gamma _2$, the shape regularity parameter $\varrho$ (3.1) of the mesh, the final time *T*, and the size of the domain $\Omega$, but not on the mesh size parameters nor the derivatives of the coefficients in *A* or $c$. The fact that the constant does not depend on the derivatives of *A* nor $c$ is crucial, since these (if they exist) are large for the problems of interest. This is sometimes also noted as *C* being independent of *the variations of* *A* *and* $c$.

We now formulate the variational form of problem (2.1). Find $u(\cdot ,t) \in V$ such that $u(\cdot ,0) = u_0$ and

2.2 $$\begin{aligned} (\dot{u},v)_c+ a(u,v) = (f,v), \quad \forall v \in V, \ t\in (0,T], \end{aligned}$$

and $$a(u,v) = (A\nabla u,\nabla v)$$.

The following theorem states existence and uniqueness of a solution to (2.2). The proof is based on Galerkin approximations, see, e.g., [5, 9].

### Theorem 2.1

Assume that (A1)–(A4) are satisfied. Then there exists a unique solution *u* to (2.2) such that $u \in L_2(0,T;H^1_0)$ and $\dot{u}\in L_2(0,T;H^{-1})$.

## Numerical approximation

In this section we describe the local orthogonal decomposition method presented in [13] to define a generalized finite element method for the multiscale problem (2.2).

First we introduce some notation. Let {${\mathcal {T}}_h\}_{h>0}$ and {${\mathcal {T}}_H\}_{H>h}$ be families of shape regular triangulations of $\Omega$ where $h_K:= {{\mathrm{diam}}}(K)$, for $K\in {\mathcal {T}}_h$, and $H_K: = {{\mathrm{diam}}}(K)$, for $K\in {\mathcal {T}}_H$. We also define $H:=\max _{K\in {\mathcal {T}}_H} H_K$ and $h:=\max _{K\in {\mathcal {T}}_h} h_K$. Furthermore, we let $\varrho >0$ denote the shape regularity parameter of the mesh ${\mathcal {T}}_H$;

3.1 $$\begin{aligned} \varrho :=\max _{K \in {\mathcal {T}}_H} \varrho _K, \ \text {with} \ \varrho _K:= \frac{{{\mathrm{diam}}}B_K}{{{\mathrm{diam}}}K}, \ \text {for}\ K\in {\mathcal {T}}_H, \end{aligned}$$

where $B_K$ is the largest ball contained in *K*.

Now define the classical piecewise affine finite element spaces

$$\begin{aligned} V_H&= \{v\in C(\bar{\Omega }): v=0 \text { on } \partial \Omega , v|_K \text { is a polynomial of degree} \le 1, \forall K \in {\mathcal {T}}_H\},\\ V_h&= \{v\in C(\bar{\Omega }): v=0 \text { on } \partial \Omega , v|_K \text { is a polynomial of degree} \le 1, \forall K \in {\mathcal {T}}_h\}. \end{aligned}$$

We let ${\mathcal {N}}$ denote the interior nodes of $V_H$ and $\varphi _x$ the corresponding hat function for $x\in {\mathcal {N}}$, such that ${{\mathrm{span}}}(\{\varphi _x\}_{x \in {\mathcal {N}}})=V_H$. We further assume that ${\mathcal {T}}_h$ is a refinement of ${\mathcal {T}}_H$, so that $V_H\subseteq V_h$. Finally, we also need the finite element mesh ${\mathcal {T}}_H$ of $\Omega$ to be such that the $L_2$-projection $P_H$ onto the finite element space $V_H$ is stable in $H^1$-norm, see, e.g., [3], and the references therein.

To discretize in time we introduce the uniform discretization

3.2 $$\begin{aligned} 0=t_0<t_1<\cdots <t_N=T, \quad \text {where } t_n-t_{n-1} = \tau . \end{aligned}$$

Let $U_n$ be the approximation of *u*(*t*) at time $t=t_n$ and denote $f_n:=f(t_n)$. Using the notation $\bar{\partial }_t U_n = (U_n-U_{n-1})/\tau$ we now formulate the classical backward Euler FEM; find $U_n\in V_h$ such that

3.3 $$\begin{aligned} (\bar{\partial }_t U_n,v)_c+ a(U_n,v) = (f_n,v), \quad \forall v \in V_h, \end{aligned}$$

for $n=1,\dots ,N$ and $U_0 \in V_h$ is some approximation of $u_0$. We also define the operator ${\mathcal {A}}_h: V_h \rightarrow V_h$ by

3.4 $$\begin{aligned} ({\mathcal {A}}_h v,w) = a(v,w), \quad \forall v, w \in V_h. \end{aligned}$$

The convergence of the classical finite element approximation (3.3) depends on $\Vert D^2u\Vert$, where $D^2$ denotes the second order derivatives. If the diffusion coefficient *A* oscillates on a scale of $\epsilon$ we may have $\Vert D^2u\Vert \sim \epsilon ^{-1}$, see [17] for a further discussion. The total error is thus typically bounded by $\Vert u(t_n)-U_n\Vert \le C(\tau + (h/\epsilon )^2)$, which is small only if $h<\epsilon$.

The purpose of the method described in this paper is to find an approximate solution, let us denote it by $\hat{U}$ for now, in some space $\hat{V} \subset V_h$, such that $\dim {\hat{V}} = \dim {V_H}$, for $H> h$, and the error $\Vert U_n-\hat{U}_n\Vert \le CH^2$. Here *C* is independent of the variations in *A* and $c$ and $\hat{U}_n$ is less expensive to compute than $U_n$. The total error is then the sum of two terms

$$\begin{aligned} \Vert u(t_n)-\hat{U}_n\Vert \le \Vert u(t_n)-U_n\Vert + \Vert U_n-\hat{U}_n\Vert , \end{aligned}$$

where the first term is the error due to the classical FEM approximation with backward Euler discretization in time. This is small if *h* (and $\tau$) is chosen sufficiently small, that is, if *h* resolves the variations of *A*. Hence, we think of $h>0$ as fixed and appropriately chosen. Our aim is now to analyze the error $\Vert U_n-\hat{U}_n\Vert$.

We emphasize that $\hat{V}=V_H$ is not sufficient. The total error would in this case typically be $\Vert u(t_n)-\hat{U}_n\Vert \sim (\tau + (H/\epsilon )^2)$, which is small only if $H<\epsilon$.

The next theorem states some regularity results for (3.3).

### Theorem 3.1

Assume that (A1)–(A4) are satisfied. Then, for $1\le n \le N$, there exists a unique solution $U_n$ to (3.3) such that $U_n \in V_h$. Furthermore, if $U_0=0$, then we have the bound

3.5 $$\begin{aligned} \Vert \bar{\partial }_t U_n\Vert \le C\left( \Vert f\Vert _{L_\infty (L_2)} + \Vert \dot{f}\Vert _{L_\infty (L_2)}\right) , \end{aligned}$$

and, if $f=0$, then

3.6 $$\begin{aligned} \Vert \bar{\partial }_tU_n\Vert \le Ct_n^{-1} \Vert U_0\Vert ,\ n \ge 1, \quad \Vert \bar{\partial }_t\bar{\partial }_tU_n\Vert \le Ct_n^{-2} \Vert U_0\Vert ,\ n\ge 2, \end{aligned}$$

where *C* depends on $\alpha _1$, $\gamma _1$, $\gamma _2$ and *T*, but not on the variations of *A* or $c$.

### Proof

From (3.3) it follows for $n\ge 2$ that

$$\begin{aligned} \left( \bar{\partial }_t \bar{\partial }_tU_n,v\right) _c+ a\left( \bar{\partial }_tU_n,v\right) = \left( \bar{\partial }_tf_n,v\right) , \quad \forall v \in V_h, \end{aligned}$$

and choosing $v=\bar{\partial }_t U_n$ we derive

$$\begin{aligned} \Vert c^{1/2} \bar{\partial }_tU_n\Vert \le \Vert c^{1/2}\bar{\partial }_tU_1\Vert + \frac{1}{\sqrt{\gamma _1}}\sum _{j=2}^n\tau \Vert \bar{\partial }_tf_j\Vert . \end{aligned}$$

From (3.3) we have, since $U_0=0$, $\Vert c^{1/2}\bar{\partial }_tU_1\Vert \le \gamma _1^{-1/2}\Vert f_1\Vert$. Finally, using the inequality

$$\begin{aligned} \sum _{j=2}^n\tau \Vert \bar{\partial }_tf_j\Vert \le \sum _{j=2}^n\max _{t_{j-1}\le \xi \le t_j}\tau \Vert \dot{f}(\xi )\Vert \le C\Vert \dot{f}\Vert _{L_\infty (L_2)}, \end{aligned}$$

and the bounds on $c$ in (A2) we deduce (3.5).

To derive the bounds in (3.6), we define the solution operator $E_{n}$ such that $E_n v$ is the solution to (3.3) with $f=0$ and initial data $v \in L_2$. Let $\{\varphi _i\}$ and $\{\lambda _i\}$ be eigenfunctions and corresponding eigenvalues such that

$$\begin{aligned} a(\varphi _i, v) = \lambda _i (\varphi _i,v)_c, \quad \forall v \in V. \end{aligned}$$

It follows that the eigenvalues $\{\lambda _i\}$ are real and positive and $\{\varphi _i\}$ are orthogonal with respect to the inner products $(\cdot ,\cdot )_c$ and $a(\cdot ,\cdot )$. Furthermore, there is a finite subset of eigenfunctions that spans $V_h$, i.e., ${{\mathrm{span}}}\{\varphi _i\}_{i=1}^M = V_h$ for some $M<\infty$. With this notation, the solution $E_n v$ can be written as

$$\begin{aligned} E_n v = \sum _{i=1}^M \frac{1}{(1+\tau \lambda _i)^n}(v, \varphi _i)_c\varphi _i. \end{aligned}$$

The bounds now follows from [18, Lemma 7.3]. $\square$

### Orthogonal decomposition

In this section we describe the orthogonal decomposition which defines the GFEM space denoted $\hat{V}$ in the discussion above. We refer to [13, 14] for details. The GFEM space is defined using only the diffusion coefficient *A*, that is, the variations in $c$ are not accounted for in the construction of the space. In Sect. 4 we prove that this space indeed is sufficient to obtain convergence of the method.

For the construction of the GFEM space we use the (weighted) Clément interpolation operator introduced in [4], ${\mathfrak {I}}_{H}:V_h \rightarrow V_{H}$ defined by

3.7 $$\begin{aligned} {\mathfrak {I}}_H v = \sum _{x\in {\mathcal {N}}}({\mathfrak {I}}_H v)(x)\varphi _x, \quad \text {where} \quad (\mathfrak {I}_H v)(x) : = \frac{\int _{\Omega } v \varphi _x}{\int _{\Omega } \varphi _x}. \end{aligned}$$

For this interpolation operator the following result is proved [4]

3.8 $$\begin{aligned} H^{-1}_K\Vert v-{\mathfrak {I}}_H v\Vert _{L_2(K)} + \Vert \nabla (v-{\mathfrak {I}}_H v)\Vert _{L_2(K)} \le C \Vert \nabla v\Vert _{L_2(\bar{\omega }_K)}, \forall v \in V, \end{aligned}$$

where $\bar{\omega }_K :=\cup \{\bar{K} \in {\mathcal {T}}_H: \bar{K} \cap K \ne \emptyset \}$ and *C* depends on the shape regularity $\varrho$.

Let $V^{\mathrm {f}} = \{v \in V_h: {\mathfrak {I}}_H v = 0\}$ be the kernel of the Clément interpolation operator (3.7). This space contains all fine scale features not resolved by $V_H$. The space $V_h$ can then be decomposed into $V_h = V_H \oplus V^\mathrm {f}$, where $v\in V_h$ can be written as a sum $v=v_H+v^\mathrm {f}$, with $v_H\in V_H$, $v^{\mathrm {f}} \in V^{\mathrm {f}}$, and $(v_H,v^{\mathrm {f}})=0$.

Now define the orthogonal projection $R^{\mathrm {f}}:V_h \rightarrow V^{\mathrm {f}}$ by

$$\begin{aligned} a(R^{\mathrm {f}}v, w) = a( v, w) \quad \forall w \in V^\mathrm {f}, \ v \in V_h. \end{aligned}$$

Using this projection we define the GFEM space, also referred to as the multiscale space,

$$\begin{aligned} V^{\mathrm {ms}} :=V_H-R^{\mathrm {f}}V_H, \end{aligned}$$

which leads to another orthogonal decomposition $V_h = V^{\mathrm {ms}} \oplus V^{\mathrm {f}}$. Hence any function $v\in V_h$ has a unique decomposition $v=v^{\mathrm {ms}} + v^{\mathrm {f}}$, with $v^{\mathrm {ms}}\in V^{\mathrm {ms}}$ and $v^{\mathrm {f}} \in V^{\mathrm {f}}$, with $a(v^{\mathrm {ms}},v^{\mathrm {f}})=0$.

To define a basis for $V^{\mathrm {ms}}$ we want to find the projection $R^{\mathrm {f}}$ of the nodal basis function $\varphi _x\in V_H$. Let this projection be denoted $\phi _x$, so that $\phi _x\in V^{\mathrm {f}}$ satisfies the (global) corrector problem

3.9 $$\begin{aligned} a(\phi _x,w) = a(\varphi _x,w), \quad \forall w\in V^{\mathrm {f}}. \end{aligned}$$

A basis for the multiscale space $V^{\mathrm {ms}}$ is thus given by

$$\begin{aligned} \{\varphi _x-\phi _x:x\in {\mathcal {N}}\}. \end{aligned}$$

We also introduce the projection $R^{\mathrm {ms}}:V_h\rightarrow V^{\mathrm {ms}}$, defined by

3.10 $$\begin{aligned} a(R^{\mathrm {ms}} v, w) = a(v,w), \quad \forall w \in V^{{\mathrm {ms}}}, \ v \in V_h. \end{aligned}$$

Note that $R^{\mathrm {ms}} = I-R^{\mathrm {f}}$. For $R^{\mathrm {ms}}$ we have the following lemma, based on the results in [13].

#### Lemma 3.2

For the projection $R^{\mathrm {ms}}$ in (3.10) and $v\in V_h$ we have the error bound

3.11 $$\begin{aligned} \Vert v-R^{\mathrm {ms}} v\Vert&\le CH^2\Vert {\mathcal {A}}_h v\Vert , \quad v \in V_h, \end{aligned}$$

where *C* depends on $\alpha _1$ and $\varrho$, but not on the variations of *A* or $c$.

#### Proof

Define the following elliptic auxiliary problem: find $z \in V_h$ such that

$$\begin{aligned} a(z,w) = (v - R^{\mathrm {ms}} v,w), \quad \forall w \in V_h. \end{aligned}$$

In [13, Lemma 3.1] it was proven that the solution to an elliptic equation of the form

$$\begin{aligned} a(u,w)=(g,w), \quad \forall w \in V_h, \end{aligned}$$

satisfies the error estimate

$$\begin{aligned} \mathopen {|||} u-R^{\mathrm {ms}} u \mathclose {|||} \le CH\Vert g\Vert , \end{aligned}$$

where *C* depends on $\varrho$ and $\alpha _1$, but not on the variations of *A*. Hence, we have the following bound for *z*,

$$\begin{aligned} \mathopen {|||} z-R^{\mathrm {ms}} z \mathclose {|||} \le CH\Vert v-R^{\mathrm {ms}} v\Vert . \end{aligned}$$

Furthermore, we note that $v-R^{\mathrm {ms}} v \in V_h$ and

$$\begin{aligned} \Vert v-R^{\mathrm {ms}} v\Vert ^2&= (v - R^{\mathrm {ms}} v,v - R^{\mathrm {ms}} v) = a(z,v - R^{\mathrm {ms}} v) \\ {}&= a(z - R^{\mathrm {ms}} z,v - R^{\mathrm {ms}} v)\le \mathopen {|||} z-R^{\mathrm {ms}} z \mathclose {|||} \; \mathopen {|||} v - R^{\mathrm {ms}} v \mathclose {|||} . \end{aligned}$$

Now, since $a(v,w)=({\mathcal {A}}_hv,w)$, we get $\mathopen {|||} v-R^{\mathrm {ms}} v \mathclose {|||} \le CH\Vert {\mathcal {A}}_h v\Vert$ and (3.11) follows. $\square$

In particular, if $U_n$ is the solution to (3.3), then (3.11) gives

$$\begin{aligned} \Vert U_n-R^{\mathrm {ms}} U_n\Vert&\le CH^2\Vert P_h(f_n-c\,\bar{\partial }_tU_n)\Vert , \quad n\ge 1,\\ \Vert \bar{\partial }_tU_n-R^{\mathrm {ms}}\bar{\partial }_t U_n\Vert&\le CH^2\Vert P_h(\bar{\partial }_t f_n-c\,\bar{\partial }_t\bar{\partial }_t U_n)\Vert , \quad n\ge 2, \end{aligned}$$

where $P_h$ is the $L_2$-projection onto $V_h$.

The result in Lemma 3.2 should be compared with the error of the classical Ritz projection $R_h:V\rightarrow V_h$ defined by $a(R_hv,w)=a(v,w)$, $\forall w \in V_h$. Using elliptic regularity estimates, one achieves

$$\begin{aligned} \Vert R_hv-v\Vert \le Ch^2\Vert D^2v\Vert \le Ch^2\Vert {\mathcal {A}}v\Vert , \end{aligned}$$

which is similar to the result in Lemma 3.2. However, in this case, *C* depends on the variations of *A* and the regularity of $\Omega$. This is avoided by using the $R^{\mathrm {ms}}$-projection, since the constant in Lemma 3.2 does not depend on the variations of *A* or *c*.

Now define the corresponding GFEM to problem (3.3); find $U^{\mathrm {ms}}_n \in V^{\mathrm {ms}}$ such that

3.12a $$\begin{aligned} (\bar{\partial }_t U^{\mathrm {ms}}_n,v)_c + a(U^{\mathrm {ms}}_n,v)&= (f_n,v), \quad \forall v \in V^{\mathrm {ms}},\end{aligned}$$

3.12b $$\begin{aligned} (U^{\mathrm {ms}}_0,v)_c&= (U_{0},v)_c, \,\, \forall v \in V^{\mathrm {ms}}, \end{aligned}$$

for $n=1,\dots ,N$. Furthermore, we define the operator ${\mathcal {A}}^{\mathrm {ms}}: V^{\mathrm {ms}} \rightarrow V^{\mathrm {ms}}$ by

3.13 $$\begin{aligned} ({\mathcal {A}}^{\mathrm {ms}} v,w) = a(v,w), \quad \forall v, w \in V^{\mathrm {ms}}. \end{aligned}$$

#### Remark 3.3

In this remark we discuss the possibilities of including time dependency in the coefficients $c$ and *A*.

(i) It is possible, with a slight modification of the error analysis, to let $c=c(x,t)$ be time dependent with rapid variations in space. However, for simplicity, we shall only study the time independent case here.
(ii) We emphasize that the construction of the method in (3.12) depends on the fact that the diffusion coefficient does not depend on time. If we have $A = A(x,t)$, the multiscale space could be updated in each time step. For each $t_n$, we would then define a new Ritz projection $R^{\mathrm {f}}$ with $A = A(x,t_n)$ leading to a space $V^{\mathrm {ms}}_n$. If the variations in time are slow or periodic, it is also possible reuse the space for several time steps. However, if the variations are fast and non-periodic, then updating the basis may become too expensive which calls for a different approach.

### Localization

Since the corrector problems (3.9) are posed in the fine scale space $V^{\mathrm {f}}$ they are computationally expensive to solve. Moreover, the correctors $\phi _x$ generally have global support, which destroys the sparsity of the resulting linear system (3.12). However, as shown in [13], $\phi _x$ decays exponentially fast away from *x*. This observation motivates a localization of the corrector problems to smaller patches of coarse elements. Here we use a variant presented in [6], which reduces the required size of the patches.

We first define the notion of patches and their sizes. For all $K \in {\mathcal {T}}_H$ we define $\omega _k(K)$ to be the patch of size *k*, where

$$\begin{aligned}&\omega _0(K) := \text {int } K,\\&\omega _k(K) := \text {int } \big (\cup \{\hat{K}\in {\mathcal {T}}_H: {\hat{K}}\cap \overline{\omega _{k-1}(K)} \ne \emptyset \}\big ), \quad k=1,2,\dots \end{aligned}$$

Moreover, we define $V^{\mathrm {f}}(\omega _k(K)) := \{w \in V^{\mathrm {f}}: v(z)=0 \text { on } \overline{\Omega }\setminus \omega _k(K)\}$.

Now define the operator $R^{\mathrm {f}}_K:V_h \rightarrow V^{\mathrm {f}}$ by

$$\begin{aligned} \int _\Omega A\nabla R^{\mathrm {f}}_Kv\cdot \nabla w = \int _K A\nabla v \cdot \nabla w, \quad \forall v \in V_h,\ w \in V^{\mathrm {f}}, \end{aligned}$$

and note that $R^{\mathrm {f}} := \sum _{K \in {\mathcal {T}}_H}R^{\mathrm {f}}_K$. We now localize the operator $R^{\mathrm {f}}_K$ by defining $R_{K,k}^{\mathrm {f}}:V_h \rightarrow V^{\mathrm {f}}(\omega _k(K))$ through

$$\begin{aligned} \int _{\omega _k(K)}A\nabla R_{K,k}^{\mathrm {f}}v\cdot \nabla w = \int _K A\nabla v \cdot \nabla w, \quad \forall v \in V_h,\ w \in V^{\mathrm {f}}(\omega _k(K)), \end{aligned}$$

and we define $R_{k}^{\mathrm {f}} := \sum _{K \in {\mathcal {T}}_H}R_{K,k}^{\mathrm {f}}$. Hence we can, for each nonnegative integer *k*, define a localized multiscale space

$$\begin{aligned} V^{\mathrm {ms}}_k:=V_H - R^{\mathrm {f}}_kV_H. \end{aligned}$$

Here the basis is given by $\{\varphi _x-\phi _{k,x}: x \in {\mathcal {N}}\}$, where $\phi _{k,x} = R_{k}^{\mathrm {f}}\varphi _x$ is the localized version of $\phi _x$. The procedure of decomposing $V_h$ into the orthogonal spaces $V^{\mathrm {ms}}$ and $V^{\mathrm {f}}$ together with the localization of $V^{\mathrm {ms}}$ to $V^{\mathrm {ms}}_k$ is referred to as *local orthogonal decomposition*.

The following lemma follows from Lemma 3.6 in [6].

#### Lemma 3.4

There exists a constant $0< \mu < 1$ that depends on the contrast $\alpha _2/\alpha _1$ such that

$$\begin{aligned} \mathopen {|||} R^{\mathrm {f}}v-R^{\mathrm {f}}_kv \mathclose {|||} \le Ck^{d/2}\mu ^{k} \mathopen {|||} v \mathclose {|||} , \quad \forall v \in V_h, \end{aligned}$$

where *C* depends on $\alpha _1$, $\alpha _2$, and $\varrho$, but not on the variations of *A* or $c$.

Now let $R^{\mathrm {ms}}_k:V_h \rightarrow V^{\mathrm {ms}}_k$ be the orthogonal projection defined by

3.14 $$\begin{aligned} a\left( R^{\mathrm {ms}}_k v, w\right) = a(v, w), \quad \forall w \in V^{\mathrm {ms}}_k. \end{aligned}$$

The next lemma is a consequence of Theorem 3.7 in [6] and estimates the error due to the localization procedure.

#### Lemma 3.5

For the projection $R^{\mathrm {ms}}_k$ in (3.14) we have the bound

3.15 $$\begin{aligned} \Vert v -R^{\mathrm {ms}}_k v\Vert&\le C\left( H+k^{d/2}\mu ^{k}\right) ^2\Vert {\mathcal {A}}_h v\Vert , \quad \forall v \in V_h. \end{aligned}$$

Here *C* depends on $\alpha _1$, $\alpha _2$, and $\varrho$, but not on the variations of *A* or $c$.

#### Proof

The proof is similar to the proof of Lemma 3.2. Let $z\in V_h$ be the solution to the elliptic dual problem

$$\begin{aligned} a(z,w)=(v - R^{\mathrm {ms}}_k v,w), \quad \forall w \in V_h, \end{aligned}$$

which gives

$$\begin{aligned} \Vert v-R^{\mathrm {ms}}_k v\Vert ^2&=\left( v-R^{\mathrm {ms}}_k v, v-R^{\mathrm {ms}}_k v\right) = a\left( z-R^{\mathrm {ms}}_k z,v-R^{\mathrm {ms}}_k v\right) \\&\le \mathopen {|||} z-R^{\mathrm {ms}}_k z \mathclose {|||} \mathopen {|||} v-R^{\mathrm {ms}}_kv \mathclose {|||} . \end{aligned}$$

It follows from Theorem 3.7 in [6] that there exists a constant *C* depending on $\alpha _2$, $\alpha _1$, and $\varrho$, such that $\mathopen {|||} z-R^{\mathrm {ms}}_k z \mathclose {|||} \le C(H+k^{d/2}\mu ^k)\Vert v-R^{\mathrm {ms}}_k v\Vert$, with $\mu$ as in Lemma 3.4. Since $({\mathcal {A}}_hv,w)=a(v,w)$ we get $\mathopen {|||} v-R^{\mathrm {ms}}_kv \mathclose {|||} \le C(H+k^{d/2}\mu ^{k})\Vert {\mathcal {A}}_hv\Vert$ and (3.15) follows. $\square$

We are now ready to formulate the localized version of (3.12) by replacing $V^{\mathrm {ms}}$ by $V^{\mathrm {ms}}_k$. The localized GFEM formulation reads; find $U^{{\mathrm {ms}}}_{k,n} \in V^{{\mathrm {ms}}}_k$ such that

3.16a $$\begin{aligned} \left( \bar{\partial }_t U^{{\mathrm {ms}}}_{k,n},v\right) _c+ a\left( U^{\mathrm {ms}}_{k,n},v\right)&= (f_n,v), \quad \forall v \in V^{\mathrm {ms}}_k,\end{aligned}$$

3.16b $$\begin{aligned} \left( U^{\mathrm {ms}}_{k,0},v\right) _c&= (U_{0},v)_c, \,\, \forall v \in V^{\mathrm {ms}}_k, \end{aligned}$$

for $n=1,\dots ,N$. We also define the operator ${\mathcal {A}}^{\mathrm {ms}}_k:V^{\mathrm {ms}}_k \rightarrow V^{\mathrm {ms}}_k$ by a localized version of (3.13)

3.17 $$\begin{aligned} \left( {\mathcal {A}}^{\mathrm {ms}}_k v,w\right) = a(v,w), \quad \forall v, w \in V^{\mathrm {ms}}_k. \end{aligned}$$

We also define the solution operator $E^{\mathrm {ms}}_{k,n}$, such that the solution to (3.16), with $f=0$, can be expressed as $U^{\mathrm {ms}}_{k,n} = E^{\mathrm {ms}}_{k,n}U^{\mathrm {ms}}_{k,0}$. For this operator we have estimates similar to (3.6). Since the initial data in (3.16) is the projection onto $V^{\mathrm {ms}}_k$ with respect to the inner product $(\cdot ,\cdot )_c$, we define $P^{\mathrm {ms}}_{c,k}:L_2\rightarrow V^{\mathrm {ms}}_k$ by

$$\begin{aligned} \left( P^{\mathrm {ms}}_{c,k}v,w\right) _c= (v,w)_c, \quad \forall w \in V^{\mathrm {ms}}_k, \end{aligned}$$

to state the next lemma.

#### Lemma 3.6

For $l=0,1$, and $v\in L_2$, we have

$$\begin{aligned} \Vert \bar{\partial }_t^l E^{\mathrm {ms}}_{k,n}P^{\mathrm {ms}}_{c,k}v\Vert \le Ct_n^{-l}\Vert v\Vert , \quad n\ge l, \quad \mathopen {|||} E^{\mathrm {ms}}_{k,n}P^{\mathrm {ms}}_{c,k}v \mathclose {|||} \le Ct_n^{-1/2}\Vert v\Vert ,\quad n\ge 1, \end{aligned}$$

where *C* depends on the constant $\alpha _1$, $\gamma _1$, $\gamma _2$, but not on the variations of *A* or $c$.

#### Proof

As in the proof of Theorem 3.1 there exist a finite number of positive eigenvalues $\{\lambda _i\}_{i=1}^M$ and corresponding orthogonal eigenvectors $\{\varphi _i\}_{i=1}^M$ such that ${{\mathrm{span}}}\,\{\varphi _i\}=V^{\mathrm {ms}}_k$ and

$$\begin{aligned} a(\varphi _i,v)=\lambda _i(\varphi _i,v)_c, \quad \forall v \in V. \end{aligned}$$

It follows that $E^{\mathrm {ms}}_{k,n}v$ can be written as

$$\begin{aligned} E^{\mathrm {ms}}_{k,n}v = \sum _{i=1}^M \frac{1}{(1+\tau \lambda _i)^n}(v,\varphi _i)_c\varphi _i, \end{aligned}$$

and the bounds now follow from [18, Lemma 7.3]. $\square$

## Error analysis

In this section we derive error estimates for the local orthogonal decomposition method introduced in Sect. 3. The bounds of the time derivatives of a parabolic problem with nonsmooth initial data, (c.f. Theorem 3.1), depends on negative powers of $t_n$, which leads to error bounds containing negative powers of $t_n$. These are non-uniform in time, but of optimal order for a fix time $t_n>0$. The same phenomenon appears in classical finite element analysis for equations with nonsmooth initial data, see [18] and references therein. The error analysis in this section is carried out by only taking the $L_2$-norm of $U_{0}$, which allows $u_0 \in L_2$.

### Theorem 4.1

Let $U_n$ be the solution to (3.3) and $U^{{\mathrm {ms}}}_{k,n}$ the solution to (3.16). Then, for $1\le n\le N$,

$$\begin{aligned} \Vert U^{{\mathrm {ms}}}_{k,n}-U_n\Vert&\le C\Big (1+\log \frac{t_n}{\tau }\Big )(H+k^{d/2}\mu ^k)^2\big (t_n^{-1}\Vert U_{0}\Vert + \Vert f\Vert _{L_\infty (L_2)} \\&\quad +\, \Vert \dot{f}\Vert _{L_\infty (L_2)}\big ), \end{aligned}$$

where *C* depends on $\alpha _1$, $\alpha _2$, $\gamma _1$, $\gamma _2$, $\varrho$, and *T*, but not on the variations of *A* or $c$.

The proof of Theorem 4.1 is divided into several lemmas. To study the error in the homogeneous case, $f=0$, we use techniques similar to the classical finite element analysis of problems with nonsmooth initial data, see [18] and the references therein.

Define $T_h:L_2\rightarrow V_h$ and $T^{\mathrm {ms}}_k:L_2\rightarrow V^{\mathrm {ms}}_k$ by

$$\begin{aligned} a(T_h v, w)&= (v,w)_c, \quad \forall w \in V_h, \, v\in L_2, \\ a\left( T^{\mathrm {ms}}_k v, w\right)&= (v,w)_c, \quad \forall w \in V^{\mathrm {ms}}_k, \, v\in L_2. \end{aligned}$$

With this notation the solution to the parabolic problem (3.3), with $f=0$, can be expressed as $T_h\bar{\partial }_t{U}_n + U_n=0$, since

$$\begin{aligned} a(T_h \bar{\partial }_t U_n, w) = (\bar{\partial }_t U_n, w)_c= -a(U_n, w), \quad \forall w\in V_h. \end{aligned}$$

Similarly, the solution to (3.16), with $f=0$, can be expressed as $T^{\mathrm {ms}}_k\bar{\partial }_t{U}^{\mathrm {ms}}_{k,n} + U^{\mathrm {ms}}_{k,n}=0$. Note that $T_h$ and $T^{\mathrm {ms}}_k$ are self-adjoint and positive semi-definite with respect to $(\cdot ,\cdot )_c$ on $L_2$, and $T^{\mathrm {ms}}_k = R^{\mathrm {ms}}_k T_h$.

Now, let $e_n=U^{\mathrm {ms}}_{k,n}-U_n$, where $e_n$ solves the error equation

4.1 $$\begin{aligned} T^{\mathrm {ms}}_k\bar{\partial }_t {e_n}+e_n=-U_n-T^{\mathrm {ms}}_k\bar{\partial }_t U_n=(T_h-T^{\mathrm {ms}}_k)\bar{\partial }_tU_n= (R^{\mathrm {ms}}_k-I) U_n=:\rho _{n}, \end{aligned}$$

for $n=1,\dots ,N$ with $T^{\mathrm {ms}}_ke_0=0$, since

$$\begin{aligned} a(T^{\mathrm {ms}}_ke_0, w) = (U^{\mathrm {ms}}_k - U_0, w)_c= 0. \end{aligned}$$

The following lemma is a discrete version of [18, Lemma 3.3].

### Lemma 4.2

Suppose $e_n$ satisfies the error equation (4.1). Then

4.2 $$\begin{aligned} \Vert e_n\Vert ^2&\le C\Big (\Vert \rho _{n}\Vert ^2 + t_n^{-1}\Big ( \sum _{j=1}^n\tau \Vert \rho _{j}\Vert ^2 + \sum _{j=2}^n \tau t_j^2\Vert \bar{\partial }_t \rho _{j}\Vert ^2\Big )\Big ), \quad n \ge 2, \end{aligned}$$

4.3 $$\begin{aligned} \Vert e_1\Vert&\le C\Vert \rho _1\Vert , \end{aligned}$$

where *C* depends on $\gamma _1$ and $\gamma _2$, but not on the variations of *A* or $c$.

### Proof

Multiply the error equation (4.1) by $c\,\bar{\partial }_t e_n$ and integrate over $\Omega$ to get

$$\begin{aligned} (T^{\mathrm {ms}}_k \bar{\partial }_te_n,\bar{\partial }_t e_n)_c+ (e_n, \bar{\partial }_t e_n)_c= (\rho _{n},\bar{\partial }_t e_n)_c, \end{aligned}$$

where the first term on the left hand side is nonnegative, since $T^{\mathrm {ms}}_k$ is positive semi-definite on $L_2$. Multiplying by $\tau t_n$ we have

$$\begin{aligned} t_n\Vert c^{1/2}e_n\Vert ^2 - t_{n}(e_n,e_{n-1})_c\le t_n(\rho _{n}, e_n - e_{n-1})_c, \end{aligned}$$

which gives

$$\begin{aligned} \frac{t_n}{2}\Vert c^{1/2} e_n\Vert ^2 - \frac{t_{n-1}}{2}\Vert c^{1/2}e_{n-1}\Vert ^2&\le t_n(\rho _{n}, e_n-e_{n-1})_c+ \frac{t_n-t_{n-1}}{2}\Vert c^{1/2}e_{n-1}\Vert ^2 \\&\le t_n(\rho _{n}, e_n)_c- t_{n-1}(\rho _{n-1}, e_{n-1})_c\\&\quad - (t_n\rho _n-t_{n-1}\rho _{n-1},e_{n-1})_c+ \frac{\tau }{2}\Vert c^{1/2}e_{n-1}\Vert ^2. \end{aligned}$$

Summing over *n* now gives

$$\begin{aligned} t_n\Vert c^{1/2}e_n\Vert ^2 - t_1\Vert c^{1/2}e_1\Vert ^2&\le 2t_n(\rho _{n}, e_n)_c- 2t_1(\rho _{1}, e_1)_c\\&\quad -\, \sum _{j=2}^n2(t_j\rho _j-t_{j-1}\rho _{j-1},e_{j-1})_c+ \sum _{j=2}^n\tau \Vert c^{1/2}e_{j-1}\Vert ^2, \end{aligned}$$

and thus,

$$\begin{aligned} t_n\Vert c^{1/2}e_n\Vert ^2&\le C\Big (t_n\Vert c^{1/2}\rho _n\Vert ^2 + \sum _{j=2}^n \tau \big (t_j^2\Vert c^{1/2}\bar{\partial }_t \rho _j\Vert ^2 + \Vert c^{1/2}\rho _{j-1}\Vert ^2\big ) \\&\quad +\, \sum _{j=2}^n\tau \Vert c^{1/2}e_{j-1}\Vert ^2\Big ). \end{aligned}$$

To estimate the last sum we note that, since $T^{\mathrm {ms}}_k$ is self-adjoint and positive semi-definite,

$$\begin{aligned} 2(T^{\mathrm {ms}}_k\bar{\partial }_te_n,e_n)_c&= (T^{\mathrm {ms}}_k\bar{\partial }_te_n,e_n)_c+ (T^{\mathrm {ms}}_ke_n, \bar{\partial }_t e_n)_c\\&= \bar{\partial }_t (T^{\mathrm {ms}}_ke_n,e_n)_c+ \tau (T^{\mathrm {ms}}_k\bar{\partial }_te_n,\bar{\partial }_te_n)_c\ge \bar{\partial }_t(T^{\mathrm {ms}}_ke_n,e_n)_c. \end{aligned}$$

so by multiplying the error equation (4.1) by $2ce_n$ we get

$$\begin{aligned} \bar{\partial }_t(T^{\mathrm {ms}}_ke_n,e_n)_c+ 2\Vert c^{1/2}e_n\Vert ^2 \le 2(T^{\mathrm {ms}}_k\bar{\partial }_te_n,e_n)_c+ 2\Vert c^{1/2}e_n\Vert ^2 = 2(\rho _n,e_n)_c. \end{aligned}$$

Multiplying by $\tau$ and summing over *n* gives

$$\begin{aligned} (T^{\mathrm {ms}}_ke_n,e_n)_c+ \sum _{j=1}^n\tau \Vert c^{1/2}e_j\Vert ^2 \le \sum _{j=1}^n \tau \Vert c^{1/2}\rho _j\Vert ^2, \end{aligned}$$

where we have used that $T^{\mathrm {ms}}_ke_0=0$. Since the first term is nonnegative we deduce that $\sum _{j=1}^n\tau \Vert c^{1/2}e_j\Vert ^2 \le \sum _{j=1}^n \tau \Vert c^{1/2}\rho _j\Vert ^2$ and (4.2) follows. For $n=1$ this also proves (4.3). Note that we have used the bounds on *c* in (A2) to obtain the result in the $L_2$-norm. $\square$

Next lemma is a discrete version of a result that can be found in the proof of [18, Theorem 3.3].

### Lemma 4.3

Under the assumptions of Lemma 4.2 we have, for $n \ge 2$, the bound

4.4 $$\begin{aligned} \Vert e_n\Vert \le Ct_n^{-1} \Big (\max _{2\le j\le n}t_j^2\Vert \bar{\partial }_t \rho _j\Vert + \max _{1\le j\le n}\Big (t_j\Vert \rho _j\Vert + \Vert \sum _{r=1}^j\tau \rho _r\Vert \Big )\Big ), \end{aligned}$$

where *C* depends on $\gamma _1$ and $\gamma _2$, but not on the variations of *A* or $c$.

### Proof

It follows from Lemma 4.2 that

$$\begin{aligned} \Vert e_n\Vert \le C\left( \max _{2\le j \le n}t_j\Vert \bar{\partial }_t\rho _j\Vert + \max _{1\le j \le n}\Vert \rho _j\Vert \right) , \quad n \ge 2, \end{aligned}$$

or by using Young’s inequality with different constants the proof can be modified to show that

$$\begin{aligned} \Vert e_n\Vert \le \epsilon \max _{2\le j \le n}t_j\Vert \bar{\partial }_t\rho _j\Vert + C(\epsilon )\max _{1\le j \le n}\Vert \rho _j\Vert , \quad n \ge 2, \end{aligned}$$

for some $\epsilon >0$. Now define $z_j=t_je_j$. Then

$$\begin{aligned} T^{\mathrm {ms}}_k\bar{\partial }_t z_n + z_n&= t_n\rho _n + T^{\mathrm {ms}}_ke_{n-1}:=\eta _n, \quad n \ge 1, \end{aligned}$$

and, since $T^{\mathrm {ms}}_k z_0 = 0$ we conclude from Lemma 4.2

$$\begin{aligned} \Vert z_n\Vert \le \epsilon \max _{2\le j \le n}t_j\Vert \bar{\partial }_t\eta _j\Vert + C\max _{1\le j \le n}\Vert \eta _j\Vert . \end{aligned}$$

From the definition of $\eta _j$ it follows that

$$\begin{aligned} \Vert \eta _j\Vert&\le t_j\Vert \rho _j\Vert + \Vert T^{\mathrm {ms}}_ke_{j-1}\Vert , \quad j \ge 1. \end{aligned}$$

Furthermore, for $$j\ge 2$$

$$\begin{aligned} t_j\Vert \bar{\partial }_t \eta _j\Vert&\le t_j\Vert \bar{\partial }_tt_j\rho _j\Vert + t_j\Vert \bar{\partial }_t T^{\mathrm {ms}}_ke_{j-1}\Vert \\&\le t_j^2 \Vert \bar{\partial }_t\rho _j\Vert +t_j \Vert \rho _{j-1}\Vert + t_j\Vert \rho _{j-1}-e_{j-1}\Vert \\&\le t_j^2 \Vert \bar{\partial }_t\rho _j\Vert + 2t_j\Vert \rho _j-\rho _{j-1}\Vert + 2t_j \Vert \rho _{j}\Vert + t_{j}\Vert e_{j-1}\Vert \\&\le 3t_j^2 \Vert \bar{\partial }_t\rho _j\Vert + 2t_j\Vert \rho _{j}\Vert + 2t_{j-1}\Vert e_{j-1}\Vert \\&\le C\Big (t_j^2\Vert \bar{\partial }_t\rho _j\Vert + t_j\Vert \rho _j\Vert \Big ) + 2\Vert z_{j-1}\Vert , \end{aligned}$$

where we used $\frac{1}{2}t_{j}\le t_{j-1} \le t_j$ for $j\ge 2$. To bound $\Vert T^{\mathrm {ms}}_ke_n\Vert$ we define $\tilde{e}_n = \sum _{j=1}^n\tau e_j$ and $\tilde{e}_0 =0$. Multiplying the error equation (4.1) by $\tau$ and summing over *n* gives

$$\begin{aligned} \sum _{j=1}^n \tau T^{\mathrm {ms}}_k \bar{\partial }_t e_j + \tilde{e}_n = T^{\mathrm {ms}}_k \bar{\partial }_t \tilde{e}_n + \tilde{e}_n = \tilde{\rho }_n, \quad n \ge 1, \end{aligned}$$

where $\tilde{\rho }_n=\sum _{j=1}^n \tau \rho _j$ and we have used that $T^{\mathrm {ms}}_ke_0=0$. Note that by definition $T^{\mathrm {ms}}_k\tilde{e}_0 = 0$. Thus, by Lemma 4.2, we have

$$\begin{aligned} \Vert \tilde{e}_n\Vert&\le C\Big (\max _{2\le j \le n}t_j\Vert \bar{\partial }_t\tilde{\rho }_j\Vert + \max _{1\le j \le n}\Vert \tilde{\rho }_j\Vert \Big )\\ {}&\le C\Big (\max _{2\le j \le n}t_j\Vert \rho _j\Vert + \max _{1\le j \le n}\Vert \sum _{r=1}^j \tau \rho _r\Vert \Big ). \end{aligned}$$

Hence, since $$T^{\mathrm {ms}}_k \bar{\partial }_t \tilde{e}_n = T^{\mathrm {ms}}_k e_n$$,

$$\begin{aligned} \Vert T^{\mathrm {ms}}_ke_n\Vert \le \Vert \tilde{e}_n\Vert + \Vert \tilde{\rho }_n\Vert \le C\Big (\max _{2\le j \le n}t_j\Vert \rho _j\Vert + \max _{1\le j \le n}\Vert \sum _{r=1}^j \tau \rho _r\Vert \Big ). \end{aligned}$$

With $\epsilon = \frac{1}{4}$ we get

$$\begin{aligned} \Vert z_n\Vert&\le \frac{1}{4} \max _{2\le j \le n}t_j\Vert \bar{\partial }_t\eta _j\Vert + C\max _{1\le j \le n}\Vert \eta _j\Vert \\&\le \frac{1}{2} \max _{1\le j \le n}\Vert z_{j}\Vert + C\Big (\max _{2\le j \le n}t_j^2\Vert \bar{\partial }_t\rho _j\Vert + \max _{1\le j \le n}(t_j\Vert \rho _j\Vert + \Vert \sum _{r=1}^j \tau \rho _r\Vert )\Big ), \end{aligned}$$

but from (4.3) we deduce $\Vert z_1\Vert \le t_1\Vert \rho _1\Vert$, and hence

$$\begin{aligned} \Vert z_n\Vert&\le \frac{1}{2} \max _{2\le j \le n}\Vert z_{j}\Vert + C\Big (\max _{2\le j \le n}t_j^2\Vert \bar{\partial }_t\rho _j\Vert + \max _{1\le j \le n}(t_j\Vert \rho _j\Vert + \Vert \sum _{r=1}^j \tau \rho _r\Vert )\Big ). \end{aligned}$$

Choosing $n^*$ such that $\max _{2\le j \le n}z_j = z_{n^*}$ we conclude (4.4). $\square$

### Lemma 4.4

Assume $f=0$ and let $U^{{\mathrm {ms}}}_{k,n}$ be the solution to (3.16) and $U_n$ the solution to (3.3). Then, for $1\le n \le N$,

$$\begin{aligned}&\Vert U^{{\mathrm {ms}}}_{k,n}-U_{n}\Vert \le C(H+k^{d/2}\mu ^{k})^2t_n^{-1}\Vert U_{0}\Vert \end{aligned}$$

where *C* depends on $\alpha _1$, $\alpha _2$, $\gamma _1$, $\gamma _2$, $\varrho$, and *T*, but not on the variations of *A* or $c$.

### Proof

From Lemma 4.3 we have

$$\begin{aligned} \Vert e_n\Vert \le Ct_n^{-1} \Big (\max _{2\le j\le n}t_j^2\Vert \bar{\partial }_t \rho _j\Vert + \max _{1\le j \le n}\Big (t_j\Vert \rho _j\Vert + \Vert \sum _{r=1}^j\tau \rho _r\Vert \Big )\Big ), \quad n \ge 2, \end{aligned}$$

and from Lemma 4.2 $\Vert e_1\Vert \le C\Vert \rho _1\Vert$. The rest of the proof is based on estimates for the projection $R^{\mathrm {ms}}_k$ in Lemma 3.5 and the regularity of the homogeneous equation (3.6). We have

$$\begin{aligned} t_j^2\Vert \bar{\partial }_t{\rho }_j\Vert&\le C\left( H+k^{d/2}\mu ^k\right) ^2 t_j^2\Vert {\mathcal {A}}_h\bar{\partial }_tU_j\Vert \\&\le C\left( H+k^{d/2}\mu ^k\right) ^2t_j^2\Vert c^{1/2}\bar{\partial }_t\bar{\partial }_tU_j\Vert \le C\left( H+k^{d/2}\mu ^k\right) ^2\Vert U_0\Vert , \quad \!\! j\ge 2,\\ t_j\Vert \rho _j\Vert&\le C\left( H+k^{d/2}\mu ^k\right) ^2 t_j\Vert {\mathcal {A}}_hU_j\Vert \\&\le C\left( H+k^{d/2}\mu ^k\right) ^2 t_j\Vert c^{1/2}\bar{\partial }_tU_j\Vert \le C\left( H+k^{d/2}\mu ^k\right) ^2\Vert U_0\Vert , \quad j \ge 1, \\ \Vert \sum _{r=1}^j \tau \rho _r\Vert&= \Vert \sum _{r=1}^j\tau \left( T_h-T^{\mathrm {ms}}_k\right) \bar{\partial }_t{U}_r\Vert \le \Vert \left( T_h-T^{\mathrm {ms}}_k\right) (U_j-U_0)\Vert \\&\le C\left( H+k^{d/2}\mu ^k\right) ^2\Vert U_0\Vert , \end{aligned}$$

where we have used $T^{\mathrm {ms}}_k=R^{\mathrm {ms}}_kT_h$ and $\Vert U_j\Vert \le C\Vert U_0\Vert$. $\square$

The next lemma concerns the convergence of the inhomogeneous parabolic problem (2.1) with initial data $U_{0}=0$.

### Lemma 4.5

Assume $U_{0}=0$ and let $U^{\mathrm {ms}}_{k,n}$ be the solution to (3.16) and $U_n$ the solution to (3.3). Then, for $1\le n \le N$,

$$\begin{aligned} \Vert U^{\mathrm {ms}}_{k,n}-U_n\Vert&\le C\left( 1 + \log \frac{t_n}{\tau }\right) \left( H+k^{d/2}\mu ^{k}\right) ^2\left( \Vert f\Vert _{L_\infty (L_2)}+\Vert \dot{f}\Vert _{L_\infty (L_2)}\right) , \end{aligned}$$

where *C* depends on $\alpha _1$, $\alpha _2$, $\gamma _1$, $\gamma _2$, $\varrho$, and *T*, but not on the variations of *A* or $c$.

### Proof

Let $U^{\mathrm {ms}}_{k,n}- U_n = U^{\mathrm {ms}}_{k,n}-R^{\mathrm {ms}}_k U_n + R^{\mathrm {ms}}_k U_n - U_n=:\theta _{n} + \rho _{n}$. For $\rho _{n}$ we use Lemma 3.5 to achieve the estimate

$$\begin{aligned} \Vert \rho _{n}\Vert \le C(H+k^{d/2}\mu ^{k})^2\Vert {\mathcal {A}}_h U_n\Vert . \end{aligned}$$

Now, for $v \in V^{\mathrm {ms}}_k$ we have

$$\begin{aligned} \left( \bar{\partial }_t \theta _{n},v\right) _c+ a(\theta _{n},v) =(-\bar{\partial }_t \rho _{n},v)_c. \end{aligned}$$

Using Duhamel’s principle we have

$$\begin{aligned} \theta _n = \tau \sum _{j=1}^n E^{\mathrm {ms}}_{k,n-j+1}P^{\mathrm {ms}}_{c,k}(-\bar{\partial }_t \rho _{j}), \end{aligned}$$

since $\theta _0=0$. Summation by parts now gives

$$\begin{aligned} \theta _n = E^{\mathrm {ms}}_{k,n}P^{\mathrm {ms}}_{c,k}\rho _0 - P^{\mathrm {ms}}_{c,k}\rho _n - \tau \sum _{j=1}^n \bar{\partial }_tE^{\mathrm {ms}}_{k,n-j+1}P^{\mathrm {ms}}_{c,k}\rho _{j}. \end{aligned}$$

Note that $\rho _0=0$. Using Lemma 3.5 and Lemma 3.6 we get

$$\begin{aligned} \Vert \theta _n\Vert&\le C\Bigg (\Vert \rho _n\Vert + \tau \sum _{j=1}^n t_{n-j+1}^{-1}\Vert \rho _{j}\Vert \Bigg )\\ {}&\le C\left( H+k^{d/2}\mu ^k\right) ^2\max _{1\le j\le n}\Vert {\mathcal {A}}_hU_j\Vert \left( 1+\tau \sum _{j=1}^nt_{n-j+1}^{-1}\right) , \end{aligned}$$

where the last sum can be bounded by

$$\begin{aligned} \tau \sum _{j=1}^nt^{-1}_{n-j+1}\le 1 + \log \frac{t_n}{\tau }. \end{aligned}$$

It remains to bound $\Vert {\mathcal {A}}_h U_n\Vert$. We have ${\mathcal {A}}_hU_n=P_h(f_n-c\bar{\partial }_tU_n)$ and Theorem 3.1 gives

$$\begin{aligned} \Vert {\mathcal {A}}_hU_j\Vert \le C(\Vert f_j\Vert + \Vert \bar{\partial }_t U_j\Vert ) \le C(\Vert f\Vert _{L_\infty (L_2)} + \Vert \dot{f}\Vert _{L_\infty (L_2)}), \end{aligned}$$

which completes the proof. $\square$

### Proof (of Theorem 4.1)

The result follows from Lemmas 4.4 and 4.5 by rewriting $U_n=U_{n,1}+U_{n,2}$, where $U_{n,1}$ is the solution to the homogeneous problem and $U_{n,2}$ the solution to the inhomogeneous problem with vanishing initial data. $\square$

### Remark 4.6

We note that the choice of *k* and the size of $\mu$ determine the rate of the convergence. In general, to achieve optimal order convergence rate, *k* should be chosen proportional to $\log (H^{-1})$, i.e. $k =c \log (H^{-1})$. With this choice of *k* we have $\Vert U^{\mathrm {ms}}_{k,n}-U_n\Vert \le C(1+\log n)H^2t_n^{-1}$.

## The semilinear parabolic equation

In this section we discuss how the above techniques can be extended to a semilinear parabolic problem with multiscale diffusion coefficient. In this section we assume, for simplicity, that the coefficient $c= 1$.

### Problem formulation

We are interested in equations of the form

5.1 $$\begin{aligned} \begin{array}{rl} \dot{u} - \nabla \cdot (A\nabla u) = f(u),&{}\quad \text {in } \Omega \times (0,T], \\ u = 0,&{}\quad \text {on } \partial \Omega \times (0,T], \\ u(\cdot ,0) = u_0, &{}\quad \text {in } \Omega , \end{array} \end{aligned}$$

where $f:{\mathbb {R}}\rightarrow {\mathbb {R}}$ is twice continuously differentiable and $\Omega$ is a polygonal/polyhedral boundary in ${\mathbb {R}}^d$, for $d\le 3$. For $d=2,3$, *f* is assumed to fulfill the growth condition

5.2 $$\begin{aligned} |f^{(l)}(\xi )|\le C(1+|\xi |^{\delta + 1 -l}), \quad \text {for } l=1,2, \end{aligned}$$

where $\delta =2$ if $d=3$ and $\delta \in [1,\infty )$ if $d=2$. Furthermore, we assume that the diffusion *A* fulfills assumption (A1) and $u_0\in V$.

#### Example 5.1

The Allen–Cahn equation $\dot{u} - \nabla \cdot (A \nabla u) = -(u^3-u)$ fulfills the assumption (5.2).

Define the ball $B_R := \{v \in V: \Vert v\Vert _{H^1} \le R\}$. Using Hölder and Sobolev inequalities the following lemma can be proved, see [12].

#### Lemma 5.2

If *f* fulfills assumption (5.2) and $u,v \in B_R$, then

$$\begin{aligned} \Vert f(u)\Vert \le C, \ \Vert f'(u)z\Vert _{H^{-1}} \le C\Vert z\Vert ,\ \Vert f'(u)z\Vert \le C\Vert z\Vert _{H^1},\ \Vert f''(u)z\Vert _{H^{-1}} \le C\Vert z\Vert , \ \end{aligned}$$

and

$$\begin{aligned} \Vert f(u)-f(v)\Vert _{H^{-1}} \le C\Vert u-v\Vert , \end{aligned}$$

where C is a constant depending on *R*.

From (5.1) we derive the variational form; find $u(t)\in V$ such that

5.3 $$\begin{aligned} (\dot{u},v) + (A\nabla u,\nabla v) = (f(u),v), \quad \forall v \in V, \end{aligned}$$

and $u(0)=u_0$. For this problem local existence of a solution can be derived given that the initial data $u_0 \in V$, see [12].

#### Theorem 5.3

Assume that (A1) and (5.2) are satisfied. Then, for $u_0\in B_R$, there exist $T_0 = T_0(R)$ and $C_1>0$, such that (5.3) has a unique solution $u \in C(0,T_0;V)$ and $\Vert u\Vert _{L_\infty (0,T_0;V)}\le C_1R$.

For the Allen–Cahn equation it is possible to find an a priori global bound of *u*. This means that for any time *T* there exists *R* such that if *u* is a solution then $\Vert u(t)\Vert _{L_\infty (H^1)}\le R$ for $t\in [0,T]$. Thus we can apply the local existence theorem repeatedly to attain global existence, see [12].

### Numerical approximation

The assumptions and definitions of the families of triangulations $\{\mathcal {T}_h\}_{h>0}$ and $\{\mathcal {T}_H\}_{H>h}$ and the corresponding spaces $V_H$ and $V_h$ remain the same as in Sect. 3. For the discretization in time we use a uniform time discretization given by

5.4 $$\begin{aligned} 0=t_0<t_1<\cdots <t_N=T_0, \quad \text {where } t_n-t_{n-1} = \tau , \end{aligned}$$

and $T_0$ is given from Theorem 5.3. With these discrete spaces we consider the semi-implicit backward Euler scheme where $U_n \in V_h$ satisfies

5.5 $$\begin{aligned} (\bar{\partial }_t U_n,v) + (A\nabla U_n,\nabla v) = (f(U_{n-1}),v), \quad \forall v \in V_h, \end{aligned}$$

for $n=1,\dots ,N$ where $U_0 \in V_h$ is an approximation of $u_0$. It is proven in [11] that this scheme satisfies the bound

$$\begin{aligned} \Vert U_n-u(t_n)\Vert \le Ct_{n}^{-1/2}(h^2+\tau ), \end{aligned}$$

if we choose, for instance, $U_0=P_hu_0$, where $P_h$ denotes the $L_2$-projection onto $V_h$. Note that *C* in this bound depends on the variations of *A*.

The following theorem gives some regularity estimates of the solution to (5.5).

#### Theorem 5.4

Assume that (A1) and (5.2) are satisfied. Then, for $U_0\in B_R$, there exist $T_0 = T_0(R)$ and $C_1>0$ such that (5.5) has a unique solution $U_n \in V_h$, for $1\le n\le N$, and $\max _{1\le n \le N}\Vert U_n\Vert _{H^1}\le C_1R$. Moreover, the following bounds hold

$$\begin{aligned} \Vert \bar{\partial }_t U_n\Vert \le Ct_n^{-1/2},\ n\ge 1, \ \ \mathopen {|||} \bar{\partial }_t U_n \mathclose {|||} \le Ct_n^{-1},\ n\ge 1, \ \ \Vert \bar{\partial }_t\bar{\partial }_t U_n\Vert \le Ct_n^{-3/2},\ n\ge 2, \end{aligned}$$

where *C* depends on $\alpha _1$, $T_0$, and *R*, but not on the variations of *A*.

#### Proof

We only prove the estimate $\Vert \bar{\partial }_t\bar{\partial }_t U_n\Vert \le Ct_n^{-3/2}$ here. The other two follow by similar arguments.

From (5.5) we get

5.6 $$\begin{aligned} (\bar{\partial }_t\bar{\partial }_t U_n,v) + a(\bar{\partial }_t U_n,v)&= (\bar{\partial }_t f(U_{n-1}),v),\quad \forall v \in V_h, \ n\ge 2, \end{aligned}$$

5.7 $$\begin{aligned} (\bar{\partial }_t^{(3)}U_n,v) + a(\bar{\partial }_t\bar{\partial }_t U_n,v)&= (\bar{\partial }_t\bar{\partial }_t f(U_{n-1}),v), \quad \forall v\in V_h, \ n \ge 3. \end{aligned}$$

Choosing $v = \bar{\partial }_t\bar{\partial }_t U_n$ in (5.7) gives

$$\begin{aligned} \frac{1}{\tau }\Vert \bar{\partial }_t\bar{\partial }_tU_n\Vert ^2 - \frac{1}{\tau }(\bar{\partial }_t\bar{\partial }_t U_{n-1},\bar{\partial }_t\bar{\partial }_t U_n) + \mathopen {|||} \bar{\partial }_t\bar{\partial }_t U_n \mathclose {|||} ^2= (\bar{\partial }_t\bar{\partial }_t f(U_{n-1}),\bar{\partial }_t\bar{\partial }_t U_{n}), \end{aligned}$$

which gives the bound

5.8 $$\begin{aligned} \Vert \bar{\partial }_t\bar{\partial }_tU_n\Vert ^2 - \Vert \bar{\partial }_t\bar{\partial }_t U_{n-1}\Vert ^2 \le C\tau \Vert \bar{\partial }_t\bar{\partial }_t f(U_{n-1})\Vert _{H^{-1}}. \end{aligned}$$

Using Lemma 5.2 we have for $\xi _j \in (\min \{U_{n-j},U_{n-(j-1)}\},\max \{U_{n-j},U_{n-(j-1)}\})$

$$\begin{aligned} \Vert \bar{\partial }_t\bar{\partial }_t f(U_n)\Vert _{H^{-1}}&= \frac{1}{\tau ^2}\Vert f'(\xi _1)(U_{n}-U_{n-1}) - f'(\xi _2)(U_{n-1}-U_{n-2})\Vert _{H^{-1}}\\&\le \frac{1}{\tau ^2}\Vert (f'(\xi _1)-f'(\xi _2))(U_{n}-U_{n-1})\Vert _{H^{-1}} \\&\quad +\,\frac{1}{\tau ^2}\Vert f'(\xi _2)(U_{n}-2U_{n-1}+U_{n-2})\Vert _{H^{-1}} \\&\le \frac{1}{\tau ^2} \Vert (\xi _1-\xi _2)(U_{n}-U_{n-1})\Vert + C\Vert \bar{\partial }_t\bar{\partial }_t U_{n}\Vert , \end{aligned}$$

Note that $|\xi _1-\xi _2|\le |U_{n-2}-U_{n-1}| + |U_{n-1}-U_{n}|$. By using Sobolev embeddings we get

$$\begin{aligned} \frac{1}{\tau ^2} \Vert (\xi _1-\xi _2)(U_{n}-U_{n-1})\Vert&\le \max _{n-1\le j \le n} 2\Vert (\bar{\partial }_tU_{j})^2\Vert \le \max _{n-1\le j \le n} 2\Vert \bar{\partial }_t U_{j}\Vert ^2_{L_4} \\&\le C\max _{n-1\le j \le n}\Vert \bar{\partial }_tU_{j}\Vert ^2_{H^1} \le Ct^{-2}_{n-1} \le Ct^{-2}_{n}, \end{aligned}$$

where we recall the bounds $\frac{1}{2}t_j\le t_{j-1}\le t_j$ for $j\ge 2$. Multiplying by $\tau t_n^4$ in (5.8) and summing over *n* gives

$$\begin{aligned} t_n^4\Vert \bar{\partial }_t\bar{\partial }_tU_n\Vert ^2&\le C\sum _{j=3}^n(\tau t_j^4\Vert \bar{\partial }_t\bar{\partial }_t f(U_{j-1})\Vert ^2_{H^{-1}} + (t_j^4-t_{j-1}^4)\Vert \bar{\partial }_t\bar{\partial }_tU_{j-1}\Vert ^2) \\ {}&\quad + t^4_2\Vert \bar{\partial }_t\bar{\partial }_tU_2\Vert ^2\\&\le C \sum _{j=3}^n \tau \big (t_j^4\Vert \bar{\partial }_t\bar{\partial }_tU_{j-1}\Vert ^2 + t_j^4t^{-4}_{j-1} + t_{j-1}^3\Vert \bar{\partial }_t\bar{\partial }_tU_{j-1}\Vert ^2\big ) \\ {}&\quad +t^4_2\Vert \bar{\partial }_t\bar{\partial }_tU_2\Vert ^2\\&\le C\sum _{j=3}^n \tau \big (t_{j-1}^4\Vert \bar{\partial }_t\bar{\partial }_tU_{j-1}\Vert ^2 +t_{j-1}^3\Vert \bar{\partial }_t\bar{\partial }_tU_{j-1}\Vert ^2\big ), \\ {}&\quad + t^4_2\Vert \bar{\partial }_t\bar{\partial }_tU_2\Vert ^2 + Ct_n \end{aligned}$$

for $n \ge 3$. Using $\Vert \bar{\partial }_tU_j\Vert \le Ct_j^{-1/2}$ for $j\ge 1$ we get

$$\begin{aligned} t^4_2\Vert \bar{\partial }_t\bar{\partial }_tU_2\Vert ^2 \le C\tau ^2(\Vert \bar{\partial }_tU_2\Vert ^2+\Vert \bar{\partial }_tU_1\Vert ^2) \le C \tau ^2(t_2^{-1} + t_1^{-1}) \le C\tau . \end{aligned}$$

Now, to bound $\sum _{j=2}^n t_j^3\Vert \bar{\partial }_t\bar{\partial }_tU_j\Vert$, we choose $v=\bar{\partial }_t\bar{\partial }_t U_n$ in (5.6) to derive

5.9 $$\begin{aligned} \Vert \bar{\partial }_t\bar{\partial }_t U_n\Vert ^2 + \frac{1}{\tau } \mathopen {|||} \bar{\partial }_t U_n \mathclose {|||} ^2 - \frac{1}{\tau } \mathopen {|||} \bar{\partial }_t U_{n-1} \mathclose {|||} ^2&\le \Vert \bar{\partial }_t f(U_{n-1})\Vert ^2. \end{aligned}$$

and with $\xi _j$ as above, we get

$$\begin{aligned} \Vert \bar{\partial }_t f(U_{n-1})\Vert = \Vert f'(\xi _2)\bar{\partial }_t U_{n-1} \Vert \le C \mathopen {|||} \bar{\partial }_t U_{n-1} \mathclose {|||} \le Ct^{-1}_{n-1}, \end{aligned}$$

where we used Lemma 5.2 and $\mathopen {|||} \bar{\partial }_t U_j \mathclose {|||} \le Ct_{j}^{-1}$ for $j \ge 1$. Multiplying (5.9) with $\tau t_n^3$ and summing over *n* gives

$$\begin{aligned} \sum _{j=2}^n\tau t_j^3\Vert \bar{\partial }_t\bar{\partial }_t U_j\Vert ^2 + t_n^3 \mathopen {|||} \bar{\partial }_t U_n \mathclose {|||} ^2&\le C \sum _{j=2}^n \left( \tau t^3_jt^{-2}_{j-1} +\left( t^3_j-t^3_{j-1}\right) \mathopen {|||} \bar{\partial }_t U_{j-1} \mathclose {|||} ^2\right) \\&\quad + t^3_{1} \mathopen {|||} \bar{\partial }_t U_{1} \mathclose {|||} ^2 \\&\le C \sum _{j=2}^n\left( \tau t_j + \tau t^2_{j-1} \mathopen {|||} \bar{\partial }_t U_{j-1} \mathclose {|||} ^2\right) + t^3_{1} \mathopen {|||} \bar{\partial }_t U_{1} \mathclose {|||} ^2 . \end{aligned}$$

Using $\mathopen {|||} \bar{\partial }_t U_j \mathclose {|||} \le Ct_j^{-1}$ for $j\ge 1$ we get

$$\begin{aligned} \sum _{j=2}^n\tau t_j^3\Vert \bar{\partial }_t\bar{\partial }_t U_j\Vert ^2 \le C\left( t_n^2 + t_n + t_1\right) \le Ct_n, \end{aligned}$$

where *C* now depends on $t_n\le T$. So we have proved

$$\begin{aligned} t_n^4\Vert \bar{\partial }_t\bar{\partial }_tU_n\Vert ^2&\le C\sum _{j=3}^n \tau t_{j-1}^4\Vert \bar{\partial }_t\bar{\partial }_tU_{j-1}\Vert ^2 + Ct_n + \tau \\&\le C\sum _{j=2}^{n-1} \tau t_{j}^4\Vert \bar{\partial }_t\bar{\partial }_tU_{j}\Vert ^2 + Ct_{n+1} \le C\sum _{j=2}^{n-1} \tau t_{j}^4\Vert \bar{\partial }_t\bar{\partial }_tU_{j}\Vert ^2 + Ct_{n}. \end{aligned}$$

Applying the classical discrete Grönwall’s lemma gives

$$\begin{aligned} t_n^4\Vert \bar{\partial }_t\bar{\partial }_tU_n\Vert ^2 \le Ct_n, \end{aligned}$$

which proves $\Vert \bar{\partial }_t\bar{\partial }_tU_n\Vert \le Ct_n^{-3/2}$ for $n\ge 3$. For $n=2$ we proved

$$\begin{aligned} t^4_2\Vert \bar{\partial }_t\bar{\partial }_tU_2\Vert ^2 \le C\tau \le Ct_2, \end{aligned}$$

which completes the proof. $\square$

We use the same GFEM space as in Sect. 3, that is, $V^{\mathrm {ms}} = V_H - R^{\mathrm {f}}V_H$ and the localized version $V^{\mathrm {ms}}_k = V_H - R^{\mathrm {f}}_kV_H$. Furthermore, for the completely discrete scheme, we consider the time discretization defined in (5.4) and the linearized backward Euler method thus reads; find $U^{\mathrm {ms}}_{k,n} \in V^{\mathrm {ms}}$ such that $U^{\mathrm {ms}}_{k,0} = P^{\mathrm {ms}}_k U_0$ and

5.10 $$\begin{aligned} \left( \bar{\partial }_tU^{\mathrm {ms}}_{k,n}, v\right) + a\left( U^{\mathrm {ms}}_{k,n},v\right) =\left( f\left( U^{\mathrm {ms}}_{k,n-1}\right) ,v\right) , \end{aligned}$$

for $n=1,\dots ,N$ where $P^{\mathrm {ms}}_k$ is the $L_2$-projection onto $V^{\mathrm {ms}}_k$.

To derive an error estimates we represent the solution to (5.10) by using Duhamel’s principle. Note that $U^{\mathrm {ms}}_{k,n}$ is the solution to the equation

$$\begin{aligned} \bar{\partial }_t U^{\mathrm {ms}}_{k,n} + \mathcal {A}^{\mathrm {ms}}_kU^{\mathrm {ms}}_{k,n} = P^{\mathrm {ms}}_kf(U^{\mathrm {ms}}_{k,n-1}), \end{aligned}$$

and by Duhamel’s principle we get

$$\begin{aligned} U^{\mathrm {ms}}_{k,n} = E^{\mathrm {ms}}_{k,n}U^{\mathrm {ms}}_{k,0} + \tau \sum _{j=1}^n E^{\mathrm {ms}}_{k,n-j+1}P^{\mathrm {ms}}_kf(U^{\mathrm {ms}}_{k,j-1}). \end{aligned}$$

Note that we use $c=1$ in the definition of the solution operator $E^{\mathrm {ms}}_{k,n}$ in Sect. 3.2.

### Error analysis

For the error analysis we need the following generalized discrete Grönwall lemma, see, e.g., [12].

#### Lemma 5.5

Let $A,B\ge 0$, $\beta _1,\beta _2 >0$, $0\le t_0<t_n\le T$, and $0\le \varphi _n \le R$. If

$$\begin{aligned} \varphi _n \le At_n^{-1+\beta _1} + B\tau \sum _{j=1}^{n-1} t_{n-j+1}^{-1+\beta _2}\varphi _j, \end{aligned}$$

then there is a constant *C* depending on *B*, $\beta _1$, $\beta _2$, and, *T*, such that,

$$\begin{aligned} \varphi _n \le CAt_n^{-1+\beta _1}. \end{aligned}$$

Next lemma states a result for ${\mathcal {A}}^{\mathrm {ms}}_k$ which is needed in the analysis. A proof of the bound can be found in [12].

#### Lemma 5.6

The following bound holds

$$\begin{aligned} \Vert ({\mathcal {A}}^{\mathrm {ms}}_k)^{-1/2}P^{\mathrm {ms}}_kf\Vert \le C \Vert f\Vert _{H^{-1}}, \quad f \in L_2, \end{aligned}$$

where *C* depends on $\alpha _1$, but not on the variations of *A*.

#### Theorem 5.7

For given $R\ge 0$ and $T_0 >0$ let $U_n$ be the solution to (5.5) and $U^{\mathrm {ms}}_{k,n}$ be the solution to (5.10), such that $U_n, U^{\mathrm {ms}}_{k,n} \in B_R$. Then, for $1\le n \le N$,

5.11 $$\begin{aligned} \Vert U^{\mathrm {ms}}_{k,n}-U_n\Vert \le C\left( H+k^{d/2}\mu ^k\right) ^2t_n^{-1/2}, \end{aligned}$$

where *C* depends on $\alpha _1$, $\alpha _2$, $\varrho$, *R*, and $T_0$, but not on the variations of *A*.

#### Proof

First we define $e_n = U^{\mathrm {ms}}_{k,n} - U_n =(U^{\mathrm {ms}}_{k,n} - R^{\mathrm {ms}}_k U_n) + (R^{\mathrm {ms}}_kU_n - U_n)=\theta _{n} + \rho _{n}$. For $\rho _{j}$ we use Lemma 3.5 to prove the bounds

$$\begin{aligned} \Vert \rho _{j}\Vert&\le C\left( H+k^{d/2}\mu ^k\right) ^2t^{-1/2}_{j}, \quad j \ge 1, \end{aligned}$$

and

$$\begin{aligned} \Vert \bar{\partial }_t \rho _{j}\Vert \le C\left( H+k^{d/2}\mu ^k\right) ^2t^{-3/2}_{j}, \quad j\ge 2. \end{aligned}$$

For $\theta _{n}$ we have

$$\begin{aligned} \theta _{n} = E^{\mathrm {ms}}_{k,n}\theta _{0} + \tau \sum _{j=1}^n E^{\mathrm {ms}}_{k,n-j+1}P^{\mathrm {ms}}_k\left( f\left( U^{\mathrm {ms}}_{k,j-1}\right) -f(U_{j-1})-\bar{\partial }_t\rho _{j}\right) . \end{aligned}$$

To bound $\Vert \theta _{k,n}\Vert$ we first assume $n\ge 2$ and use summation by parts for the first part of the sum. Defining $n_2$ to be the integer part of *n* / 2 we can write

$$\begin{aligned} - \tau \sum _{j=1}^{n_2}E^{\mathrm {ms}}_{k,n-j+1}P^{\mathrm {ms}}_k\bar{\partial }_t\rho _j&= E^{\mathrm {ms}}_{k,n}P^{\mathrm {ms}}_k\rho _{0} - E^{\mathrm {ms}}_{k,n-n_2}P^{\mathrm {ms}}_k\rho _{n_2} \\&\quad -\, \tau \sum _{j=1}^{n_2}\bar{\partial }_tE^{\mathrm {ms}}_{k,n-j+1}P^{\mathrm {ms}}_k\rho _{j}, \end{aligned}$$

and $\theta _{n}$ can be rewritten as

$$\begin{aligned} \theta _{n}&= E^{\mathrm {ms}}_{k,n}P^{\mathrm {ms}}_ke_0 - E^{\mathrm {ms}}_{k,n-n_2}P^{\mathrm {ms}}_k\rho _{n_2} - \tau \sum _{j=1}^{n_2}\bar{\partial }_tE^{\mathrm {ms}}_{k,n-j+1}P^{\mathrm {ms}}_k\rho _{j} \\&\quad -\tau \sum _{j=n_2+1}^nE^{\mathrm {ms}}_{k,n-j+1}P^{\mathrm {ms}}_k\bar{\partial }_t\rho _{j} \\&\quad + \tau \sum _{j=1}^n({\mathcal {A}}^{\mathrm {ms}}_k)^{1/2}E^{\mathrm {ms}}_{k,n-j+1}(\mathcal {A}^{\mathrm {ms}}_k)^{-1/2}P^{\mathrm {ms}}_k(f(U^{\mathrm {ms}}_{k,j-1})-f(U_{j-1})), \end{aligned}$$

where we note that $P^{\mathrm {ms}}_ke_0=0$. To estimate these terms we need the following bounds for $\beta _1,\beta _2>0$

$$\begin{aligned} \tau \sum _{j=1}^nt_{n-j+1}^{-1+\beta _1}t_j^{-1+\beta _2} \le C_{\beta _1,\beta _2}t_n^{-1+\beta _1 + \beta _2}, \quad \tau \sum _{j=1}^{n_2}t_{n-j+1}^{-\beta _1}t_j^{-1+\beta _2} \le C_{\beta _1,\beta _2}t_n^{-\beta _1 + \beta _2}. \end{aligned}$$

see [11]. Using Lemma 3.6 we get

$$\begin{aligned} \Vert \theta _n\Vert&\le \Vert \rho _{n_2}\Vert + C\tau \sum _{j=1}^{n_2}t^{-1}_{n-j+1}\Vert \rho _{j}\Vert + C\tau \sum _{j=n_2+1}^n\Vert \bar{\partial }_t\rho _{k,j}\Vert \\&\quad + C\tau \sum _{j=1}^n t^{-1/2}_{n-j+1}\Vert f(U^{\mathrm {ms}}_{k,j-1})-f(U_{j-1})\Vert _{H^{-1}}, \end{aligned}$$

and together with Lemmas 3.5 and 5.2 this gives

$$\begin{aligned} \Vert \theta _n\Vert&\le C(H+k^{d/2}\mu ^{k})^2\Big (t_{n_2}^{-1/2} + \tau \sum _{j=1}^{n_2}t^{-1}_{n-j+1}t_{j}^{-1/2} + \tau \sum _{j=n_2+1}^nt_j^{-3/2}\Big ) \\&\quad +C\tau \sum _{j=1}^n t^{-1/2}_{n-j+1}\Vert U^{\mathrm {ms}}_{k,j-1}-U_{j-1}\Vert \\&\le C(H+k^{d/2}\mu ^{k})^2t_n^{-1/2} + C\tau \sum _{j=1}^nt^{-1/2}_{n-j+1}\Vert e_{j-1}\Vert . \end{aligned}$$

Now consider $\theta _{1}$. We can rewrite

$$\begin{aligned} \theta _{1}&= E^{\mathrm {ms}}_{k,1}\theta _{0} + \tau E^{\mathrm {ms}}_{k,1}P^{\mathrm {ms}}_k(f(U^{\mathrm {ms}}_{k,0})-f(U_0)-\bar{\partial }_t\rho _{1}) \\&=E^{\mathrm {ms}}_{k,1}P^{\mathrm {ms}}_ke_{0} - E^{\mathrm {ms}}_{k,1}P^{\mathrm {ms}}_k\rho _{1} + \tau E^{\mathrm {ms}}_{k,1}P^{\mathrm {ms}}_k(f(U^{\mathrm {ms}}_{k,0})-f(U_0)), \end{aligned}$$

and using similar arguments as above

$$\begin{aligned} \Vert \theta _{1}\Vert&\le C\left( H+k^{d/2}\mu ^k\right) ^2t_1^{-1/2} + \tau t_1^{-1/2}\Vert e_0\Vert , \end{aligned}$$

Hence, we arrive at the estimate

$$\begin{aligned} \Vert e_n\Vert \le Ct_n^{-1/2}\left( H+k^{d/2}\mu ^k\right) ^2 + C\tau \sum _{j=1}^nt^{-1/2}_{n-j+1}\Vert e_{j-1}\Vert , \quad n \ge 1, \end{aligned}$$

and we can use Lemma 5.5 to conclude (5.11). $\square$

## Numerical results

In this section we present two numerical examples to verify the predicted error estimates presented for the linear problem in Sect. 4 and the semilinear problem in Sect. 5. In both cases the domain is set to the unit square $\Omega =[0,1]\times [0,1]$ and $T = 1$. The domain $\Omega$ is discretized with a uniform triangulation and the interval [0, *T*] is divided into subintervals of equal length.

**Fig. 1:**
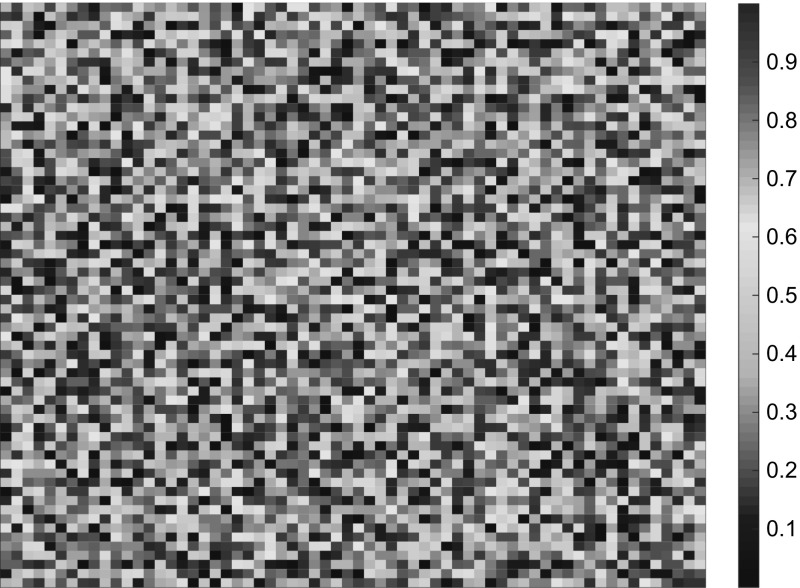
A plot of a coefficient that is piecewise constant on a Cartesian grid of size $2^{-6}$

The method is tested on two different problems. One with constant coefficients

$$\begin{aligned} c_1(x) = 1, \quad A_1(x) = \begin{pmatrix} 1 &{}\quad 0 \\ 0 &{}\quad 1 \end{pmatrix}, \end{aligned}$$

and one with multiscale coefficients

$$\begin{aligned} c_2(x) = D(x), \quad A_2(x) = \begin{pmatrix} B(x) &{}\quad 0 \\ 0 &{}\quad B(x) \end{pmatrix}, \end{aligned}$$

where *B* and *D* are piecewise constant with respect to a uniform Cartesian grid of size $2^{-6}$, see Fig. 1 for a plot of a typical coefficient. This choice of *B* and *D* imposes significant multiscale behavior on the coefficients. We expect quadratic convergence in the space of classical finite element with piecewise linear and continuous polynomials (P1-FEM) when $A=A_1$ and $c=c_1$, but poor convergence when $A=A_2$ and $c=c_2$. For the GFEM we expect quadratic convergence in both cases. Note that in the semilinear case we have $c=c_1$ in both examples.

We compute the localized GFEM in (3.16) and (5.10), denoted $U^{\mathrm {ms}}_{k,n}$, for 5 different values of the coarse grid width, $H= \sqrt{2}\cdot 2^{-2}, \sqrt{2}\cdot 2^{-3}, \sqrt{2}\cdot 2^{-4}, \sqrt{2}\cdot 2^{-5},$ and $\sqrt{2}\cdot 2^{-6}$. The time step is chosen to $\tau =0.01$ for all problems. The reference mesh ${\mathcal {T}}_h$ is of size $h=\sqrt{2}\cdot 2^{-7}$ and defines the space $V_h$ on which the localized corrector problems $\phi _{k,x}$ are solved. To measure the error, the solution $U_{n}$ in (3.3) is computed using P1-FEM on the finest scale $h=\sqrt{2}\cdot 2^{-7}$ with $\tau =0.01$.

Note that this experiment measures the error $\Vert U_{n}-U^{\mathrm {ms}}_{k,n}\Vert$. The total error $\Vert u(t_n)-U^{\mathrm {ms}}_{k,n}\Vert$ is also affected by the difference $\Vert u(t_n)-U_{n}\Vert$, which is dominating for the smaller values of *H*. We now present the results in two separate sections.

### Linear parabolic problem

For the linear parabolic problem (2.1) the right hand side is set to $f(x,t)=t$, which fulfills the assumptions for the required regularity. For simplicity the initial data is set to $u_0=1$. To construct *B* and *D* we choose, for each cell in the Cartesian grid, a value from the interval $[10^{-1},10^3]$. Note that we choose different values for *B* and *D*. This procedure gives both *B* and *D* rapidly varying features, see Fig. 1.

**Fig. 2:**
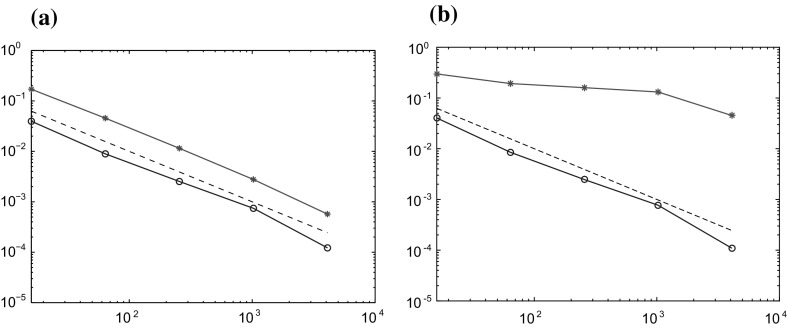
Relative $L_2$ errors $\Vert U^{\mathrm {ms}}_{k,N}-U_{h,N}\Vert /\Vert U_{h,N}\Vert$ (*blue circle*) and $\Vert U_{H,N}-U_{h,N}\Vert /\Vert U_{h,N}\Vert$ (*red asterisk*) for the linear parabolic problem plotted against the number of degrees of freedom $|{\mathcal {N}}|\approx H^{-2}$. The *dashed line* is $H^2$. **a** Constant coefficients $c_1$ and $A_1$. **b** Multiscale coefficients $c_2$ and $A_2$ (color figure online)

For each value of *H* the localized GFEM, $U^{\mathrm {ms}}_{k,n}$, and the corresponding P1-FEM, denoted $U_{H,n}$, are computed. The patch sizes *k* are chosen such that $k \sim \log (H^{-1})$, that is $k=1,2,2,3,$ and 4, for the five simulations. When computing $U_{H,n}$ the stiffness matrix is assembled on the fine scale *h* and then interpolated to the coarser scale. This way we avoid quadrature errors. The convergence results for the two examples are presented in Fig. 2, where the error at the final time $t_N$ is plotted against the degrees of freedom $|{\mathcal {N}}|$. Comparing the plots we can see the predicted quadratic convergence for the localized GFEM. Note that even though the multiscale features of $c$ are not included in the construction of the multiscale space we get convergence without pre-asymptotic effects, as suggested by the theory. However, as expected, the P1-FEM shows poor convergence on the coarse grids when the coefficients have multiscale features. We clearly see the pre-asymptotic effects when *H* does not resolve the fine structure of *B*.

### Semilinear parabolic problem

For the semilinear problem we study the Allen–Cahn equation, which has right hand side $f(u) = -(u^3-u)$ that fulfills the necessary assumptions. We define the initial data to be $u_0(x,y)=x(1-x)y(1-y)$, which is zero on $\partial \Omega$. The matrix *B* constructed as in the linear case but with values varying between $10^{-3}$ and 1. Note that, for simplicity, we have $c=1$ in both cases.

**Fig. 3:**
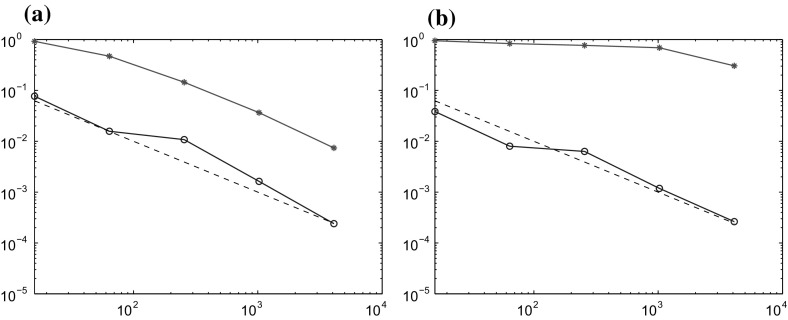
Relative $L_2$ errors $\Vert U^{\mathrm {ms}}_{k,N}-U_{h,N}\Vert /\Vert U_{h,N}\Vert$ (*blue circle*) and $\Vert U_{H,N}-U_{h,N}\Vert /\Vert U_{h,N}\Vert$ (*red asterisk*) for the semilinear parabolic problem plotted against the number of degrees of freedom $|{\mathcal {N}}|\approx H^{-2}$. The *dashed line* is $H^2$. **a** Constant coefficient $A_1$. **b** Multiscale coefficient $A_2$ (color figure online)

As in the linear case, we now compute the localized GFEM approximations $U^{\mathrm {ms}}_{k,n}$ and the corresponding P1-FEM, $U_{H,n}$. The patch sizes are chosen to $k=1,2,2,3,$ and 4, for the five simulations. The convergence results for the two examples are presented in Fig. 3. We can draw the same conclusions as in the linear case. The localized GFEM shows predicted quadratic convergence in both cases, but P1-FEM shows poor convergence on the coarse grids when the coefficients have multiscale features.
